# Solubility of Hybrid Halide Perovskites in DMF and DMSO

**DOI:** 10.3390/molecules26247541

**Published:** 2021-12-13

**Authors:** Andrey A. Petrov, Artem A. Ordinartsev, Sergey A. Fateev, Eugene A. Goodilin, Alexey B. Tarasov

**Affiliations:** 1 Laboratory of New Materials for Solar Energetics, Faculty of Materials Science, Lomonosov Moscow State University, 1 Lenin Hills, 119991 Moscow, Russia; basolon@gmail.com (A.A.P.); ordinartsev2000@yandex.ru (A.A.O.); saf1al@yandex.ru (S.A.F.); goodilin@yandex.ru (E.A.G.); 2 Department of Chemistry, Lomonosov Moscow State University, 1 Lenin Hills, 119991 Moscow, Russia

**Keywords:** hybrid perovskites, lead halide perovskites, perovskite photovoltaics, solution processing, solubility, donor numbers

## Abstract

Solution methods remain the most popular means for the fabrication of hybrid halide perovskites. However, the solubility of hybrid perovskites has not yet been quantitively investigated. In this study, we present accurate solubility data for MAPbI_3_, FAPbI_3_, MAPbBr_3_ and FAPbBr_3_ in the two most widely used solvents, DMF and DMSO, and demonstrate huge differences in the solubility behavior depending on the solution compositions. By analyzing the donor numbers of the solvents and halide anions, we rationalize the differences in the solubility behavior of hybrid perovskites with various compositions, in order to take a step forward in the search for better processing conditions of hybrid perovskites for solar cells and optoelectronics.

## 1. Introduction

Hybrid lead–halide perovskites with a general formula APbX_3_ (A = CH_3_NH_3_^+^, CH(NH_2_)_2_^+^; X = I^−^, Br^−^, Cl^−^) represent a new perspective class of materials for solar cells and optoelectronics as they possess a set of outstanding properties such as high absorption coefficients, high charge carrier mobility and intense luminescence [1,2,3]. Because of their high solubility in polar coordinating solvents, hybrid perovskites can be easily obtained using simple and cheap solutions methods such as spin-coating, slot-die coating, blade-coating, etc. [4,5]. Dimethylformamide (DMF), dimethyl sulfoxide (DMSO) and gamma-butyrolactone (GBL) are the most frequently used solvents to obtain hybrid perovskites, either in a form of polycrystalline films or as single crystals [5,6].

Although perovskite solutions with concentrations >1 M are commonly used, the solubility of single precursors, as well as the perovskites with different compositions, are almost never reported. In particular, only fragmentary data are present such as the solubility behavior of bromide perovskites in DMF and iodide perovskites in GBL, reported by Saidaminov et al. [7], which raises a question about the character of solubility behavior in other perovskite-solvent systems. Another consequence of the absence of reliable solubility data of perovskites in DMF, DMSO or mixed solvents is neglecting the fact that solutions used for perovskite processing prepared at elevated temperatures are, in fact, often oversaturated when deposited, which leads to reduced reproducibility and weak morphology control when processing light-absorbing layers [8].

Currently, despite the widespread use of perovskite solutions for almost a decade, almost no systematic studies on the solution chemistry have been reported [9]. Moreover, particular interactions between solvents and solutes have not been a subject of thorough investigation until very recently. The configurations of solvated iodoplumbate complexes in DMSO and DMF solutions of MAPbI_3_ were modeled in [10,11]. In 2020, Tutantsev et al. proposed a model explaining the interaction and relative solubility of hybrid perovskites in different solvents and showed that it relies on strong donor−acceptor, ion−dipole and hydrogen bonding interactions which can be described by donor numbers, dipole moments and Hansen hydrogen bonding parameters, respectively [12].

Herein, we present for the first time a complete set of solubility data for MAPbI_3_, FAPbI_3_, MAPbBr_3_ and FAPbBr_3_ perovskites in DMF and DMSO, and rationalize the observed differences in solubility by analyzing the donor numbers of the solvents and halide anions.

## 2. Results and Discussion

**Figure 1:**
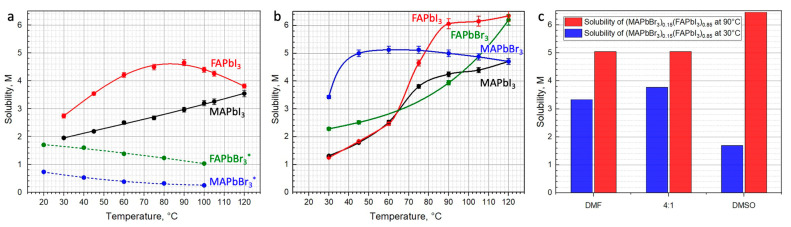
Solubility of hybrid perovskites of different compositions in DMF (**a**) and DMSO (**b**) within the range of 30–120 °C. Solubility of (MAPbBr_3_)_0.15_(FAPbI_3_)_0.85_ at 30 °C and 90 °C in DMF, DMSO and DMF/DMSO (4:1 *v*/*v*) mixture (**c**). * Data on FAPbBr_3_ and MAPbBr_3_ solubilities showed by dashed lines are determined by M. Saidaminov et al. [7].

We found that the solubilities of iodide perovskites, MAPbI_3_ and FAPbI_3_, in DMF steadily increase upon heating from 30 °C to 90 °C, demonstrating direct solubility behavior (Figure 1a). In contrast, the solubilities of bromide perovskites, MAPbBr_3_ and FAPbBr_3_, decrease upon heating (so called inverse solubility behavior) [7] (Figure 1a). At temperatures above 90 °C, the solubility of MAPbI_3_ in DMF continues to grow, whereas that of FAPbI_3_ drops significantly.

In DMSO, the solubilities of three perovskites MAPbI_3_, FAPbI_3_ and FAPbBr_3_ grow in the whole measured temperature range of 30–120 °C, while the solubility of MAPbBr_3_ demonstrate a steep increase upon heating up to 45 °C, reaching 5 M and then slowly decreases above 75 °C (Figure 1b).

The observed differences in the solubility behavior on solution composition can be explained by considering the processes associated with the solid–liquid phase equilibrium, which includes: (1) the crystal lattice breakdown of the solid phase that can release the solvent molecules if the solid phase was a solvate, and (2) the formation of haloplumbate complexes with a specific configuration of the first coordinating sphere of Pb^2+^. The latter, in turn, includes a complex equilibrium between halide ions and solvent molecules for the coordination with Pb^2+^ ions and entropy change associated with the number of solvent molecules bound to the haloplumbate complexes, such as [Pb(Hal)_n_(S)_6−n_]^(n−2)−^ (Hal = I^−^, Br^−^, Cl^−^; S = GBL, DMSO, DMF).

While the crystal lattice energy of the perovskites increases in the series MAPbI_3_–MAPbBr_3_–MAPbCl_3_ according to the experimental thermodynamic data [13], the intensity of the interaction of solvent molecules with Pb^2+^ ions can be described by donor numbers (DN), as it was shown in [12]. The donor number is determined on the basis of the experimental enthalpies of complexation with a strong electron-pair acceptor SbCl_5_, defined for the chosen solvents as DN(GBL) = 18.0, DN(DMF) = 26.6 and DN(DMSO) = 29.8 [14]. Similarly, to characterize the intensity of the interaction of halide ions with Pb^2+^ in the solutions, the donor numbers of I^−^, Br^−^ and Cl^−^ can be considered, as determined in the study [15]: DN(I^−^) = 28.9, DN(Br^−^) = 33.7 and DN(Cl^−^) = 36.2. Although the comparison of the relative donor numbers of the solvents with the donor numbers of the halide anions cannot be carried out directly, the relative changes in DN in the series of solvents or halides clearly elucidates solubility regularities and can explain the character of solubility curves for hybrid perovskites with various compositions, also considering their different crystal lattice energies and their roughly equal-entropy growth after dissolving (Table 1).

The smallest donor numbers among DMSO, DMF and GBL are observed for GBL (DN = 18.0). On the one hand, this value seems to be large enough for the solvent to compete with iodide anions (DN(I^−^) = 28.9) and to bind directly with Pb^2+^ in haloplumbate complexes. This leads to the solubility of MAPbI_3_ and FAPbI_3_ > 1.5 M. On the other hand, pure PbI_2_ is not soluble in GBL [16] which indicates that the GBL donor number is not large enough to effectively solvate PbI_2_ molecules. Furthermore, GBL cannot compete with bromide and chloride ions (DN(Br^−^) = 33.7, DN(Cl^−^) = 36.2) and as a result, more stable bromide and chloride perovskites are insoluble in GBL [13].

The maximum MAPbI_3_ solubility in GBL is the result of the co-existence of different solid phases in equilibrium with a saturated solution. The heating of the solution from 20 °C to 60 °C leads to the decomposition of ordered clusters and the release of solvent molecules into the solution; therefore, providing a large gain in entropy [16]. Above 60 °C, a high-entropy cubic perovskite phase occurs in the equilibrium with the solution, thus changing the behavior of the solubility curve. It may be assumed that the solubility of FAPbI_3_ in GBL should also exhibit a maximum due to the same reason of T < 20 °C being shifted with respect to the solubility maximum of MAPbI_3_ in GBL (60 °C) because of a higher stability of FAPbI_3_.

In general, retrograde solubility occurs when the perovskite phase coexists with the solution, as a result of lead ion desolvation or the decomposition of polynuclear haloplumbate complexes. This leads to an increase in the number of molecules in such reactions; therefore, the entropy factor shifts the equilibrium toward the less-solvated lead in the solution along with increasing the temperature. Therefore, this leads to a lower solubility of the perovskite phases, especially in the case of small donor numbers of solvents and high lattice energy of perovskites.

The higher donor number of DMF (DN = 26.6) allows it to compete with iodide anions (DN = 28.9) as well as with bromide anions (DN = 33.7) for coordination with Pb^2+^. As a result, DMF provides an increase in solubility for MAPbI_3_ and FAPbI_3_ up to 90 °C, and then retrograde solubility for bromide counterparts MAPbBr_3_ and FAPbBr_3_ (Figure 1b).

Notably, the solubility of FAPbI_3_ raises almost two-times faster with a temperature increase as compared with the solubility of MAPbI_3_, which agrees with the difference in composition of the solvate phases assumed to be in equilibrium with the solution. The formamidinium solvate phase FAPbI_3_·2DMF contains two-times the amount of solvent molecules compared to the methylammonium solvate MAPbI_3_·DMF; therefore, it leads to a higher entropy gain in the former case [18].

The most interesting situation is observed in the case of the solutions of hybrid perovskites in DMSO (DN = 29.8). The solubilities of MAPbI_3_ and FAPbI_3_ increase expectedly with temperature and demonstrate a steeper slope, indicating a higher dependency on temperature than in DMF. For bromide perovskite MAPbBr_3_, a broad maximum is observed in the range of 60–75 °C, whereas for FAPbBr_3_, the solubility increases in the whole investigated temperature range of 30–120 °C.

Significantly higher solubility of MAPbBr_3_ in DMSO as compared with FAPbBr_3_ in DMSO can be explained by the absence of solvate phases in the former, while for the latter FAPbBr_3_·DMSO adduct is known [19]. The presence of a solvate phase in the case of high concentrations typical for hybrid perovskite solutions [18,20,21] significantly reduces the amount of free solvent that solvates complexes and ions in the solution, thus decreasing the solubility. For example, equal molar ratios of solvent to solid phase are reached for the 5 M solution of MAPbBr_3_ ([DMSO]/[MAPbBr_3_] = 2.82) and 3.7 M solution of FA-based solvate ([DMSO]/[FAPbBr_3_·DMSO] = 2.82).

Importantly, one should note that the solubilities of FAPbI_3_ and MAPbBr_3_ in DMSO intersect at ~80 °C. This fact is of great importance with respect to the technological applications of solutions with mixed compositions, such as (MAPbBr_3_)_1−x_(FAPbI_3_)_x_, as they draw great attention because of the better performance and stability of the obtained materials [22]. While solvates are known to crystallize at lower temperatures [23,24], solution processing at higher temperatures by using a doctor-blade or a slot-die should thoroughly consider the difference in solubility of MAPbI_3_ and MAPbBr_3_ (Figure 1b) precipitating directly from such solutions. Therefore, the noted solubility difference starts to play an important role in this type of technologically relevant deposition technique.

To further understand the effect of mixed compositions on the total solubility of the perovskite, we also measured the solubility of one of the best compositions for perovskite solar cells (MAPbBr_3_)_0.15_(FAPbI_3_)_0.85_ at 30 °C and 90 °C [22,25]. In practice, mixed DMSO–DMF solvent is commonly used; therefore, we also tested the solubility of (MAPbBr_3_)_0.15_(FAPbI_3_)_0.85_ in the mixture of DMF and DMSO (4:1 *v*/*v*).

The results (Figure 1c) show that the solubility of (MAPbBr_3_)_0.15_(FAPbI_3_)_0.85_ in DMF at 30 °C reaches 3.3 M which is higher than that for the pure components FAPbI_3_ (2.7 M) and MAPbBr_3_ (0.6 M). Contrastingly, the solubility of (MAPbBr_3_)_0.15_(FAPbI_3_)_0.85_ in DMSO at 30 °C (1.7 M) lies between the solubilities of FAPbI_3_ (1.2 M) and MAPbBr_3_ (3.4 M). At 90 °C, the solubility of (MAPbBr_3_)_0.15_(FAPbI_3_)_0.85_ in DMF is also much higher than that for the pure components reaching 5 M, whereas it is almost the same in DMSO as the solubility of FAPbI_3_ (6.4 M). The solubility of (MAPbBr_3_)_0.15_(FAPbI_3_)_0.85_ in a mixed DMF/DMSO solvent was found to be slightly larger than that in pure DMF (3.8 M at 30 °C and 5 M at 90 °C).

The observed behavior of the solubility for mixed systems can be explained as follows: (1) for a solvent with a high DN (DMSO), which dominates in the complexation equilibrium, a change in the halogen composition does not change the forms of complexes in the solution significantly; therefore, the solubility does not increase, and (2) for DMF, as a solvent with a lower DN, an increase in iodide content leads to the formation of mixed-ligand complexes; therefore, the solubility increases significantly, especially in comparison with bromide systems.

In addition, we found that PbI_2_ solubility in the DMF/DMSO mixture (4:1 *v*/*v*) at 30 °C, 60 °C and 90 °C reaches 1.47 M, 2.90 M and 4.44 M, respectively, demonstrating a steep linear trend. This result indicates that the 1.5 M PbI_2_ solutions widely used in the two-step process are oversaturated if coated below 30 °C, which is usually the case [26].

In summary, the solutions formed by a solvent with a relatively large donor number and a perovskite with a relatively high donor number of the halide anions demonstrate a direct solubility behavior. A relative increase in the halide donor number leads to the inverse solubility behavior (iodide perovskites in GBL, bromide perovskite in DMF, chloride perovskites in DMF/DMSO) when also taking into account the difference in the lattice energies of perovskites with different compositions. Eventually, solvents with lower donor numbers (i.e., GBL) are unable to dissolve hybrid perovskites with halides of higher donor numbers (MAPbBr_3_, FAPbBr_3_, MAPbCl_3_ and FAPbCl_3_) which simultaneously have stronger interactions in a solid state. Relatively small donor numbers of a solvent, with respect to the donor number of a halide of a corresponding perovskite, lead to the insolubility of such perovskite phases, while a relatively high donor number of the halide anions would result in direct solubility behavior.

## 3. Materials and Methods

Methylammonium iodide (CH_3_NH_3_I = MAI, Dyesol, Queanbeyan, Australia), formamidinium iodide (CH_5_N_2_I = FAI, Dyesol, Queanbeyan, Australia), methylammonium bromide (CH_3_NH_3_Br = MABr, Dyesol, Queanbeyan, Australia), formamidinium bromide (CH_5_N_2_Br = FABr, Dyesol, Queanbeyan, Australia), lead iodide (PbI_2_, 99.999%, Lanhit, Moscow, Russia), lead bromide (PbBr_2_, 99.999%, Lanhit, Moscow, Russia), dimethyl sulfoxide (DMSO, anhydrous, >99.9%, Sigma-Aldrich, Saint Louis, MO, USA) and dimethylformamide (DMF, anhydrous, >99.8%, Sigma-Aldrich, Saint Louis, MO, USA) were commercially purchased.

All solutions were prepared in a glovebox under an argon atmosphere. PbI_2_, PbBr_2_, MAI, FAI, MABr and FABr were dissolved in a desired solvent (DMSO or DMF). The obtained mixtures were stirred in glass vials hermetically closed under the argon atmosphere using a magnetic stirrer, while keeping the temperature constant using a silicone oil bath for several hours until large pieces of precipitant completely disappeared forming a homogeneous colloid. After that, small amounts of a solvent (5–20 μL) were gradually added until the precipitant disappeared completely. Following each addition of a solvent, the mixture was vigorously stirred for at least 30 min. If the amount of a precipitant decreased, the solution was stirred for 30 more minutes. Otherwise, a new portion of a solvent was added.

## 4. Conclusions

In this study, the solubility for MAPbI_3_ and FAPbI_3_ in DMF and the solubility of MAPbI_3_, FAPbI_3_, MAPbBr_3_ and FAPbBr_3_ in DMSO are presented for the first time. The solubilities of MAPbI_3_ and FAPbI_3_ in DMF were found to increase with temperature up to 90 °C, unlike MAPbBr_3_ and FAPbBr_3_ which demonstrate the retrograde solubility behavior and lower solubility values. The solubilities of MAPbI_3_, FAPbI_3_ and FAPbBr_3_ in DMSO are found to increase with temperature while the solubility of MAPbBr_3_ demonstrates a maximum at 60–75 °C and slightly decreases upon further temperature increase. Solution processing at higher temperatures should thoroughly consider the differences in the solubility of FAPbI_3_ and MAPbBr_3_ precipitating directly from such solutions, as well as the retrograde solubility of these compounds in DMF and DMSO, respectively. In general, we can conclude that the main factor determining the solubility and its behavior for the selected perovskite is the equilibrium solution—solid phase. In the case of the equilibrium of the solution with the corresponding solvate, an increase in solubility with temperature is observed, which is more significant for solvates containing a greater number of solvent molecules. On the contrary, in the absence of solvate phases, the solubility decreases with a temperature increase. In the case of mixed halide systems, these equilibria are further complicated by the simultaneous competition of complexation between the two solvents and halide ions, which leads to a nonmonotonic behavior of solubility with a change in the halogen composition. As a result, the solubility of (MAPbBr_3_)_0.15_(FAPbI_3_)_0.85_ in mixed solvents demonstrates a complex behavior which should be carefully considered.

## Figures and Tables

**Table 1:** Solubility behavior of hybrid perovskite depending on the solution composition.

		Interaction Intensity Pb^2+^—X^−^				
		Iodides DN(I^−^) = 28.9		Bromides DN(Br^−^) = 33.7		Chlorides DN(Cl^−^) = 36.2
		MAPbI_3_	FAPbI_3_	MAPbBr_3_	FAPbBr_3_	MAPbCl_3_
**Interaction** **intensity Pb^2+^—S**	**GBL** **DN = 18.0**	direct + inverse [7]	inverse [7]	insoluble	insoluble	insoluble
	**DMF** **DN = 26.6**	direct	direct + inverse	inverse [7]	inverse [7]	inverse (DMF:DMSO mixture) [17]
	**DMSO** **DN = 29.8**	direct	direct	direct + inverse	direct	

“Direct” and “inverse” refer to direct (growth) and inverse (decline, retrograde) dependences of solubility on temperature.
